# Photoacoustic imaging of clofazimine hydrochloride nanoparticle accumulation in cancerous vs normal prostates

**DOI:** 10.1371/journal.pone.0219655

**Published:** 2019-07-15

**Authors:** Joel W. Y. Tan, Mikhail D. Murashov, Gus R. Rosania, Xueding Wang

**Affiliations:** 1 Department of Biomedical Engineering, University of Michigan, Ann Arbor, Michigan, United States of America; 2 Department of Pharmaceutical Sciences, University of Michigan, Ann Arbor, Michigan, United States of America; 3 Department of Radiology, University of Michigan Medical School, Ann Arbor, Michigan, United States of America; Wayne State University, UNITED STATES

## Abstract

Prostate cancer was the most common form and had the second highest death rate of male cancer in the United States in 2015. Current diagnosis techniques, such as prostate-specific antigen tests, transrectal ultrasound scans, and biopsies, are often inconclusive, and in the latter case, invasive. Here, we explore the use of clofazimine hydrochloride nanoparticles (CFZ-HCl NPs), a repurposed formulation from an FDA-approved antimycobacterial agent, as a photoacoustic contrast agent for the evaluation of prostate cancer due to its macrophage-targeting capabilities and high optical absorbance at 495 nm. Using a transgenic adenocarcinoma of the mouse prostate (TRAMP) mouse model, our results indicate a preferential accumulation of intravenously injected CFZ-HCl NPs in cancerous prostates over normal prostates. Differences in accumulation of CFZ-HCl NPs between cancerous and normal prostates were determined using a two-wavelength unmixing technique via *ex vivo* photoacoustic imaging. Thus, intravenous injection of CFZ-HCl NPs leads to differences in the interactions of the particles with cancerous vs normal prostates, while allowing for photoacoustic detection and analysis of prostate cancer. These findings could lead to the development of a new noninvasive technique for the detection and monitoring of prostate cancer progression in an animal model that can potentially be translated to human patients.

## Introduction

Prostate cancer was the leading form of cancer among men (183,529 new cases) and had the second highest death rate for cancer among men in the United States (28,848 deaths), as reported in 2015 [1]. For many years, prostate-specific antigen (PSA) blood tests and digital rectal examinations (DREs) have been the first line of detection for prostate cancer. While PSA tests act as an indicator for the stage and prognosis of the prostate cancer, PSA levels can vary significantly depending on factors such as age, lifestyle, and other medication [2–4]. Therefore, this test is rarely used on its own as a definitive indicator of prostate cancer. Abnormal readings in the PSA levels or suspicious nodules found through DREs will usually be followed up with a transrectal ultrasound scan (TRUS), which is usually complimented with a biopsy due to the fact that TRUS by itself has a low sensitivity and specificity [5, 6]. However, prostate biopsies are invasive procedures that can cause potential complications and have significant false-negative rates (e.g. 15%–31% for the traditional sextant core biopsies) [7–13]. Magnetic resonance imaging (MRI) has also been used in the assessment of prostate cancer [14–17]. However, patients with prostates that are suspected of being cancerous through MRI assessment still typically require a biopsy for confirmation of the prostate cancer [14, 15, 18]. Thus, there is a need to develop novel techniques for detecting prostate cancers through noninvasive means.

In this study, we explored the differences in the interaction of clofazimine hydrochloride nanoparticles (CFZ-HCl NPs) [19] with normal and tumor bearing prostates, using the transgenic adenocarcinoma of the mouse prostate (TRAMP) animal model [20]. CFZ is a red-pigmented dye and a weakly basic, FDA-approved, orally administered, antimycobacterial agent that is recommended by the World Health Organization as a treatment for leprosy and multidrug resistant tuberculosis [21–29]. In patients, CFZ exhibits atypical pharmacokinetic properties that result in accumulation and stabilization in tissue macrophages of solid drug biocrystals that resemble a hydrochloride (HCl) salt form of the drug [30–35]. These CFZ-HCl biocrystals were determined to be biocompatible, stable, long-lived, relatively non-toxic, and anti-inflammatory [36, 37], which led to the development of a biomimetic formulation of CFZ-HCl NPs for parenteral administration [19]. Importantly, Murashov et al. demonstrated that these CFZ-HCl NPs preserved the majority of characteristics of CFZ-HCl biocrystals, including targeting and accumulating inside macrophages *in vitro* and *in vivo* [19]. Thus, the presence of tumor associated macrophages (TAM), including those in prostate tumors [38, 39], make CFZ-HCl NPs a potentially useful targeting agent for prostate cancer detection.

In addition to the functional and therapeutic properties, CFZ-HCl NPs have also been shown to exhibit a strong signal in the Cy5 fluorescence range (650 nm excitation/670 nm emission) and peak optical absorbance at 495 nm [19, 40]. In contrast to the free dye CFZ, CFZ-HCl NPs have a redshifted peak optical absorbance at 495 nm (compared to 450 nm for free dye CFZ), making it more suitable for biological optical applications due to the reduced interference from endogenous chromophores, such as blood at this longer wavelength [41]. This strong optical absorbance makes it a suitable contrast agent to be used with photoacoustic (PA) imaging, a rapidly emerging biomedical imaging modality that combines both optical and ultrasound imaging. PA imaging relies on the PA effect, that is the generation of acoustic waves through the absorption of electromagnetic energy [42, 43]. Typically, visible or near-infrared light from a pulsed laser is used, where the energy from the pulsed laser is absorbed by the chromophores in the biological sample. This leads to thermoelastic expansion, which is then detected via an ultrasound transducer. As such, the PA signal is directly correlated with the optical absorption of the chromophores in the sample. Notably, we have previously demonstrated the capabilities of CFZ as a PA contrast agent for potential applications in arthritis [41].

In this study, we hypothesized that intravenous (IV) administration of the biomimetic formulation of CFZ-HCl NPs will result in differences in distribution of the particles in cancerous prostates and in normal prostates, which will be measurable via PA imaging. By correlating the PA signals with histopathology and quantitative drug analysis of nanoparticle distribution in the prostates, our results clearly showed a higher accumulation of CFZ-HCl NPs in cancerous prostates over normal prostates, which led to greater PA signals using a multiple wavelength PA image analysis technique. Thus, CFZ-HCl NPs may offer a noninvasive probe using PA imaging for the detection and longitudinal assessment of prostate cancer progression in an animal model with potential biomedical relevance in humans.

## Methods

### IV injection of CFZ-HCl NP in mice

Animal care was provided by the Unit for Laboratory Animal Medicine (ULAM) and all procedures on live animals were performed in accordance with institutional guidelines and approved by the Institutional Animal Care and Use Committee (IACUC) at the University of Michigan (PRO00007593; 5 May 2017). Euthanasia was performed with carbon dioxide, followed by removal of the heart.

A total of 9 mice were divided into the following 3 groups of mice (n = 3 per group): (1) Diluent injected TRAMP mice (19 weeks, Strain 008215, Jackson Laboratory, Bar Harbor, ME, USA), (2) CFZ-HCl NP injected normal mice (19 weeks, C57BL/6, Jackson Laboratory, Bar Harbor, ME, USA), and (3) CFZ-HCl NP injected TRAMP mice (19 weeks, Strain 008215, Jackson Laboratory, Bar Harbor, ME, USA). The time indicated was the age of the mice at the point of euthanasia. For the diluent injected mice (Group 1), a total of 0.3 mL of diluent with 0 mg/mL CFZ-HCl NP was IV injected into each mouse. Briefly, the diluent for the IV injectable formulation was made using polysorbate 80 (0.5% w/v, 59924 Sigma-Aldrich, St. Louis, MO, USA); sodium chloride (BP358, Fisher Scientific, Fair Lawn, NJ, USA) for isotonicity; and Milli-Q water. The pH was adjusted to pH 5 using 0.01 M HCl or 0.01 M NaOH to ensure the stability of CFZ-HCl NPs in the formulation. The diluent was sterilized by sterile filtration with a syringe filter (09-719A; 0.22 μm, MCE, Sterile; Fisher Scientific, Fair Lawn, NJ, USA). The full details of the diluent formulation have been published previously [19].

For the CFZ-HCl NP injected mice (Groups 2 and 3), a total of 0.3 mL of 19–23 mg/mL CFZ-HCl NP was IV injected into each mouse, with the concentration of CFZ-HCl NP scaled according to the weight of the individual mice (a total CFZ-HCl NP dose of 200 mg/kg). The full details of the CFZ-HCl NP formulation have been published previously [19]. This dose was selected as it represents an equivalent amount to 3–4 weeks of oral feeding performed in previous studies which allowed for sufficient accumulation of the drug in the organs [40]. After 24 hours, the mice were sacrificed, and the prostates were harvested. Each prostate was separated into two halves: one half was snap frozen in liquid nitrogen for drug quantification, and the other half was embedded in Tissue-Plus Optimal Cutting Temperature (OCT) compound (4585, Fisher HealthCare, Houston, TX, USA) for histopathology.

### Quantification of CFZ-HCl NP in tissues

Quantification of CFZ-HCl NPs in tissues was performed using a previously published protocol with some modifications [40]. Briefly, the harvested prostate was thawed, weighed, cut, and homogenized by sonication and mechanical homogenizer (Pro200; Pro Scientific, Oxford, CT) in Pierce radioimmunoprecipitation assay (RIPA) buffer (89900; Thermo Scientific, Rockford, IL, USA). Homogenates were then filtered through Pierce tissue strainers (87791; 250 μm, Thermo Scientific, Rockford, IL, USA) utilizing the gentle centrifugation (200 x g at 4°C for 5 min). The lipophilic tissue fraction was extracted with xylenes (CAS 1330-20-7/100-41-4; Fisher Chemical, Fair Lawn, NJ, USA) in triplicates, followed by the second extraction in triplicates with 9 M H_2_SO_4_ of diprotonated CFZ from the xylenes extract. Samples were centrifuged (2,000 x g at 4°C for 10 minutes) to facilitate layer separation during extractions. After acid fractions were collected, the volumes were recorded, and CFZ-HCl NP concentrations were determined spectrophotometrically. The absorbance of the supernatants was measured at λ = 540 nm (A_540_) and 750 nm (A_750_) using a Synergy-2 plate reader (Biotek Instruments). Corrected absorbance (A_540_—A_750_) was used to determine CFZ-HCl NP content via a standard curve of standards in 9 M H_2_SO_4_, and the concentration values were corrected for organ weight. To correct for extraction yield, known amounts of CFZ were added to the prostate sample before extractions; these samples were processed and analyzed concurrently with the test samples. For prostate tissues, the extraction yield averaged 83%.

### Histopathology

Histopathology was performed using a previously published protocol with some modifications [19]. In brief, the frozen tissue blocks were sectioned (6 μm thick) using a Leica 3050S cryostat, and fixed in 4% paraformaldehyde (15710, Electron Microscopy Sciences, Hatfield, PA, USA) for 10 min. The samples were incubated with Hoechst 33342 solution (H3570; 1 μM stock; 1:10,000 dilution in PBS; Life Technologies, Carlsbad, CA, USA) for 10 min at room temperature for nuclear detection. After staining was complete, sections were mounted with a drop of ProLong Gold antifade reagent (P36930, Life Technologies, Carlsbad, CA, USA) and sealed with a cover slip. For the hematoxylin and eosin (HE) staining, the sectioning and staining were performed by the In-Vivo Animal Core of ULAM at the University of Michigan. Brightfield and fluorescence images were acquired as described in the section “Brightfield and Fluorescence Microscopy”.

### Brightfield and fluorescence microscopy

Microscopy was performed using a Nikon Eclipse Ti inverted microscope (Nikon Instruments, Melville, NY, USA) as previously described [19, 40, 44]. Briefly, brightfield images were captured using the Nikon DS-3 camera (Nikon Instruments, Melville, NY, USA), and fluorescence imaging in DAPI channel (350/405 nm, blue) and Cy5 channel (640/670 nm, far-red) was performed with the Photometrics CoolSnap MYO camera system (Photometrics, Tuscon, AZ, USA) under the control of Nikon NIS-Elements AR software (Nikon Instruments, Melville, NY, USA). Illumination for fluorescence imaging was provided by the X-Cite 120Q Widefield Fluorescence Microscope Excitation Light Source (Excelitas Technology, Waltham, MA, USA).

### Sensitivity of PA imaging of CFZ-HCl NP

The CFZ-HCl NPs were prepared for concentrations of 0, 0.001, 0.003, 0.01, 0.03, 0.1, 0.3, and 1 mg/mL. Each concentration was mixed with whole blood to generate a total of 10% blood by volume. 100 μL of each concentration was injected into a polyvinyl chloride tubing (Z280348, Sigma Aldrich, St. Louis, MO, USA). The portion of the tubing containing the CFZ-HCl NPs were submerged under water to allow for acoustic coupling with a 128-element ultrasound probe with frequency range of 7 to 15 MHz (CL15-7, Philips, Andover, MA, USA). The ultrasound probe was placed parallel to the longitudinal section of the tube and connected to a commercially available research ultrasound platform (Vantage 256, Verasonics, Redmond, WA, USA) for data acquisition. A tunable pulsed laser (Surelite OPO Plus, Continuum, Santa Clara, CA, USA) pumped by the third harmonic of an Nd:YAG laser (Surelite, Continuum, Santa Clara, CA, USA) was used to generate the PA signal. The laser has tunable wavelengths between 410–680 nm and 710–2500 nm, a pulse duration of 5 ns, and a firing frequency of 10 Hz. The tube samples were imaged at a wavelength of 500 nm and averaged 20 times, with a total of 3 replicates performed.

### CFZ-HCl NP imaging via PA multiple wavelength unmixing

The CFZ-HCl NPs were prepared for concentrations of 0, 0.003, 0.03, and 0.3 mg/mL, with the concentrations chosen based on the PA sensitivity results. An identical setup to the previous section was used with the exception that the tube samples were imaged at two wavelengths of 500 nm and 584 nm, and averaged 50 times. The wavelengths of 500 nm and 584 nm were chosen as these are the known isosbestic points of oxyhemoglobin (HbO_2_) and deoxyhemoglobin (Hb), the two main chromophores in blood. The selection of the isosbestic points of blood prevented the need to distinguish between HbO_2_ and Hb, allowing for the analysis of the total hemoglobin (THb) concentration instead. If blood and CFZ-HCl NPs are assumed to be the main chromophores generating the PA signal, the following matrix equation can be used to determine the concentrations of CFZ-HCl NPs and THb.

$$k\left[\begin{array}{cc} \varepsilon_{THb@500nm} & \varepsilon_{CFZ-HCl\;NP@500nm}\\ \varepsilon_{THb@584nm} & \varepsilon_{CFZ-HCl\;NP@584nm} \end{array}\right]\left[\begin{array}{c} \left[THb\right]\\ \left[CFZ-HClNP\right] \end{array}]=[\begin{array}{c} PA_{500nm}\\ PA_{584nm} \end{array}\right]$$ (1)

Here, *k* is a constant dependent on the light fluence, the Grüneisen parameter of the sample, and the sensitivity of the imaging system. It is assumed that the light fluence, and subsequently *k*, is wavelength independent after calibration of the laser output energy. PA_λnm_ is the photoacoustic signal at λ nm, [THb] and [CFZ-HCl NP] are the concentrations of the total hemoglobin and CFZ-HCl NPs respectively, ε_THb@λnm_ and ε_CFZ-HCL NP@λnm_ are the optical extinction coefficients of hemoglobin and CFZ-HCl NPs at a wavelength of λ nm respectively. Matrix Eq (1) can then be rearranged to obtain the following matrix equation.

$$k[\begin{array}{c} \left[THb\right]\\ \left[CFZ-HCl\;NP\right] \end{array}]\;=\;{\left[\begin{array}{cc} \varepsilon_{THb@500nm} & \varepsilon_{CFZ-HCl\;NP@500nm}\\ \varepsilon_{THb@584nm} & \varepsilon_{CFZ-HCl\;NP@584nm} \end{array}\right]}^{-1}[\begin{array}{c} {PA}_{500nm}\\ {PA}_{584nm} \end{array}]$$ (2)

With the optical extinction coefficients being known, and PA_λnm_ being the PA signal measured, the right side of the matrix equation is completely known, allowing for the concentrations of [THb] and [CFZ-HCl NP] to be separately identified, albeit modified by the constant k. Hence, it should be noted that the concentrations measured are only relative concentrations, as an absolute concentration measurement would require a calibration measurement and the value of *k* to be identified, the latter of which is not a trivial task.

### *Ex vivo* PA imaging of prostate samples

**Fig 1:**
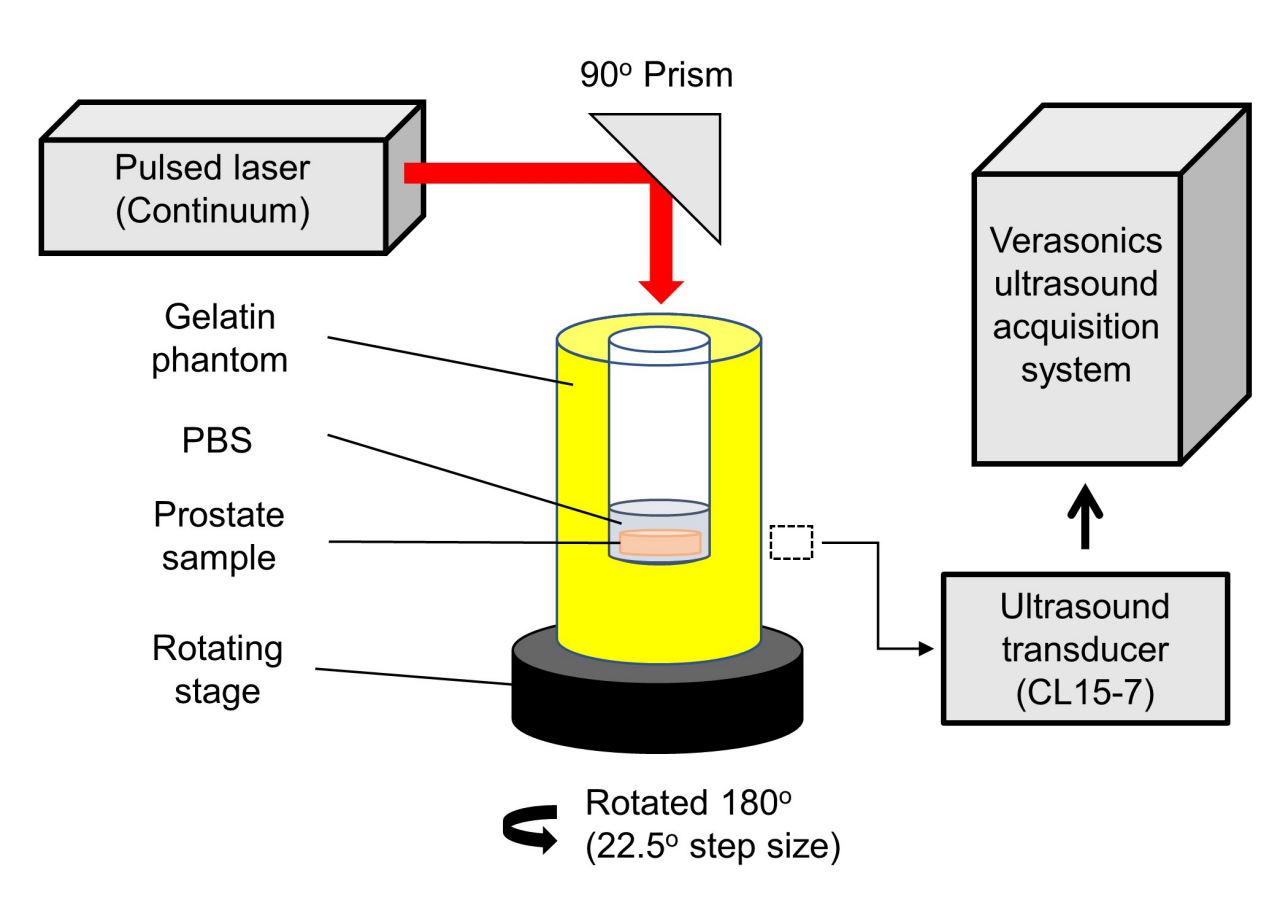
PA imaging setup for *ex vivo* prostate imaging. Dashed box indicates placement location of the ultrasound transducer (CL15-7).

The PA imaging setup is shown in Fig 1. The gelatin phantom was made using a concentration of 8 g/mL of gelatin from porcine skin (G2500, Sigma-Aldrich, St. Louis, MO, USA). The gelatin was poured into a mold to form a hollow cylindrical center and allowed to solidify. The prostate was placed in the center of the phantom, and 300 μL of PBS (pH 7.4) was added to submerge the prostate for acoustic coupling with the transducer. The gelatin phantom was placed on a calibrated rotating stage that allows for precise rotations of the gelatin phantom. The ultrasound transducer (CL15-7, Philips, Andover, MA, USA) was then placed perpendicular to the gelatin phantom at the height of the prostate sample. Ultrasound gel was added in between the transducer and the gelatin phantom for complete acoustic coupling. The ultrasound transducer was connected to a commercially available research ultrasound platform (Vantage 256, Verasonics, Redmond, WA, USA) for data acquisition. A tunable pulsed laser (Surelite OPO Plus, Continuum, Santa Clara, CA, USA) pumped by the third harmonic of an Nd:YAG laser (Surelite, Continuum, Santa Clara, CA, USA) was used to generate the PA signal. A top illumination approach was used as the prostate sample was thinner on its z-axis, allowing for a more uniform laser energy distribution throughout the prostate sample. Fifty PA images were obtained at each angle, for two wavelengths of 500 nm and 584 nm, respectively. This was repeated for 9 angles, for a total of 180° of rotation. The 50 images were then averaged, and a simple back-projection reconstruction was performed to obtain the final image at each wavelength. The PA imaging system’s resolution is determined by the ultrasound transducer (CL15-7), with an approximately 226 μm lateral and 166 μm axial resolution at a depth of 6mm. However, due to the multiple angle imaging, the resolution is improved to the better of the two resolutions, at approximately 166 μm for both lateral and axial resolutions.

The multiple wavelength unmixing algorithm previously specified was then performed to obtain the THb and CFZ-HCl NP relative concentrations. The CFZ-HCl NP signal was then normalized to the THb signal. This step was necessary as the prostate samples had different thicknesses and geometries, leading to different laser fluence distributions in the prostate samples depending on the imaging plane being measured. Mathematically, the constant “k” in matrix Eqs (1) and (2) is affected by this difference in laser fluences. However, by taking the ratio of CFZ-HCl NP/THb concentration, this constant is eliminated (with the assumption that k is wavelength-independent). This ensures that accurate comparisons can be made between prostate samples independent of their sizes. To obtain the final images, a number of image processing steps were taken. First, any normalized CFZ-HCl NP signal with a corresponding THb signal below the background signal of the THb image was removed. Next, an upper threshold was applied to the normalized CFZ-HCl NP signal. These two steps were performed to remove any extreme normalized signals (i.e. very low THb signals results in extremely high normalized CFZ-HCl NP signals). A lower threshold was also applied to the normalized CFZ-HCl NP signal to remove the background CFZ-HCl NP signal and enhance the dynamic range of the images. Finally, the image was then smoothed with a Gaussian filter. The thresholds and filters applied were identical for all images.

### Statistics

All statistical analysis was performed using Matlab R2015b (MathWorks, Natick, MA, USA). Where relevant, the data are expressed as the mean +/- standard deviation. Significant differences were determined using two-tailed two sample t-tests (*ttest2* function) and a one-way analysis of variance (ANOVA) with Tukey’s honest significant difference criterion (*anova1* followed by *multcompare* function).

## Results

### CFZ-HCl NP accumulation in prostates

To determine CFZ-HCl NP accumulation in the prostates, the mice were divided into 3 groups. The first group consisted of TRAMP mice that were IV injected with the nanoparticle diluent to act as a negative control, and the second and third groups consisted of normal and TRAMP mice, respectively, that were IV injected with CFZ-HCl NPs. While CFZ is traditionally an orally fed drug, it requires several weeks of oral CFZ free base administration for a sufficient amount of drug to accumulate in the tissue macrophages of various organs [40]. However, as a diagnosis technique, a shorter time frame would make it a more promising method for diagnosis of prostate cancer. Thus, a biomimetic formulation of CFZ-HCl NPs, which has been previously determined to be suitable for IV administration [19], allowed us to achieve a high accumulation of CFZ-HCl NPs in the cancerous prostates within 24 hours post injection.

**Fig 2:**
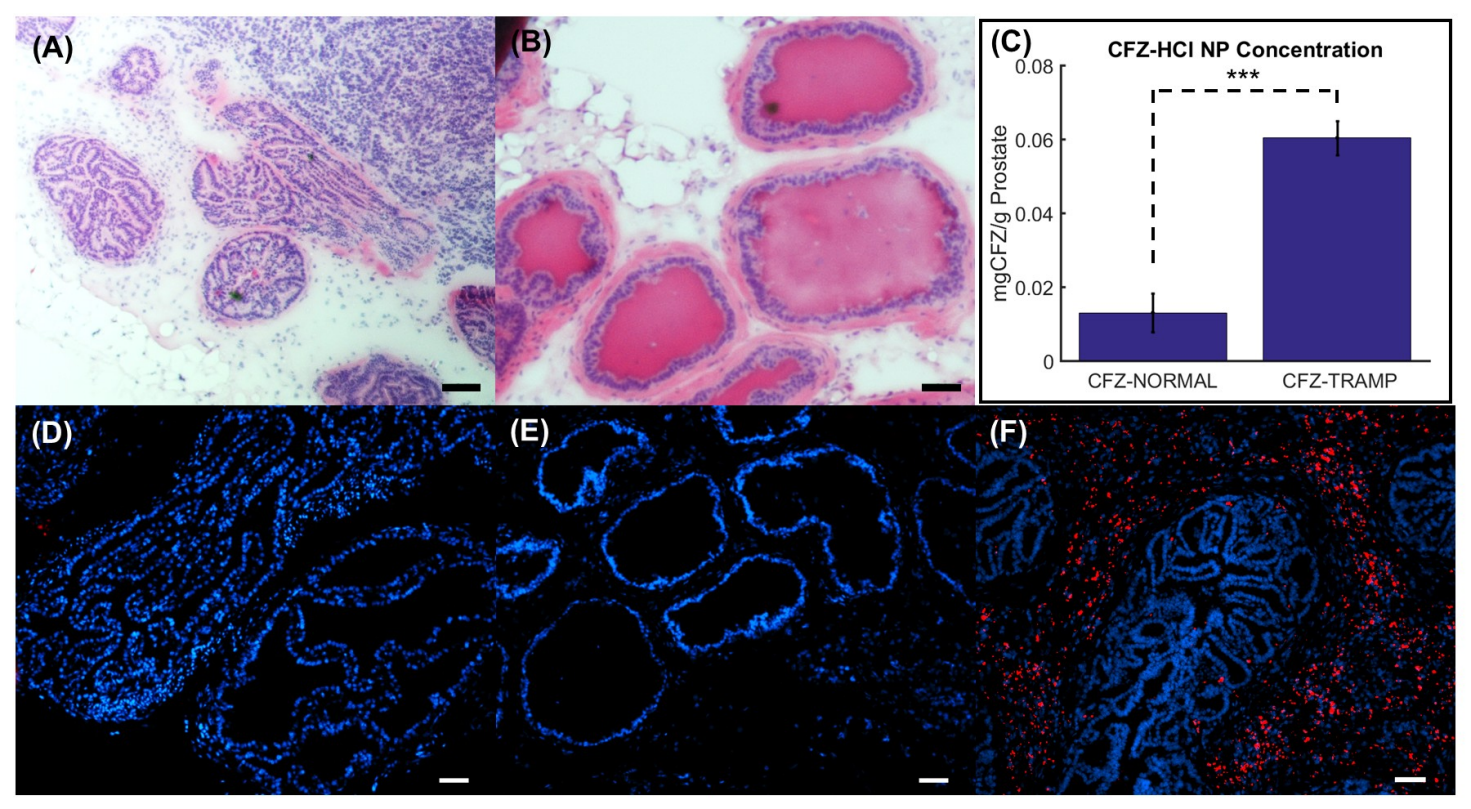
Histopathology and CFZ-HCl NP quantification in prostates. HE stained sections of the prostate for (A) TRAMP mice, and (B) Normal mice at 19 weeks. (C) Quantified concentrations of CFZ-HCl NPs in the two CFZ-HCl NP injected groups. Co-registered fluorescence images of the prostate with a nuclear stain (DAPI) in blue and CFZ-HCl NPs (Cy5) in red for (D) diluent injected TRAMP mice, (E) CFZ-HCl NP injected normal mice, and (F) CFZ-HCl NP injected TRAMP mice. Scale bar = 50 μm. *** p-value < 0.001.

HE stained sections of the TRAMP prostate (Fig 2A) showed that the mouse prostates were most likely in between the initial and advanced stages of adenocarcinoma, as the acinar lumens and interductal spaces were almost completely lost, even though the structure of each acinus remained intact [20]. The normal prostate showed a normal physiology with a uniform layer of epithelial cells comprising the glands (Fig 2B). From a quantitative drug analysis of the prostates from CFZ-HCl NP injected animals, we observed that there was a much larger CFZ-HCl NP accumulation in the TRAMP prostates than in the normal prostates (Fig 2C), verified by histopathology and fluorescence images. Furthermore, we did not observe any CFZ-HCl NP accumulation in the prostates of diluent injected TRAMP mice (Fig 2D) or in the prostates of CFZ-HCl NP injected normal mice (Fig 2E). However, there was clear accumulation of the CFZ-HCl NPs in the prostates of CFZ-HCl NP injected TRAMP (Fig 2F). These results supported the hypothesis that the IV injected CFZ-HCl NP has a greater accumulation in the TRAMP prostates than in the normal prostates.

### Sensitivity of PA imaging of CFZ-HCl NP

**Fig 3:**
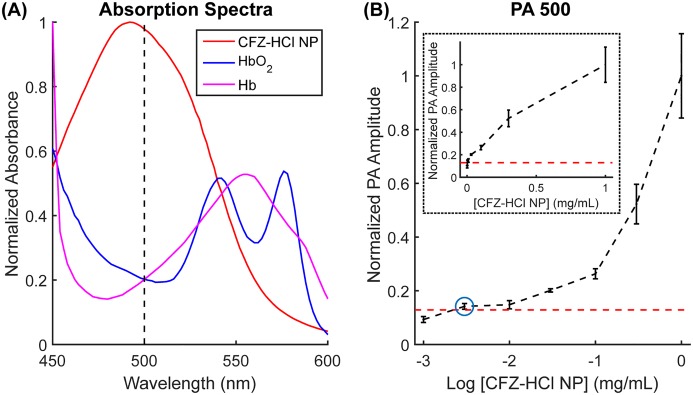
Absorption spectra and single-wavelength PA sensitivity of CFZ-HCl NP in the presence of 10% blood. (A) Overlaid absorption spectra of CFZ-HCl NP, HbO_2_, and Hb. (B) Normalized PA amplitude at 500 nm for a sample with increasing [CFZ-HCl NP] in the presence of 10% blood on a logarithmic scale. Red dotted line indicates the minimum detection threshold based on the PA measurement on a sample of 10% blood in the absence of CFZ-HCl NP. Blue circle indicates the minimum detectable CFZ-HCL NP concentration of 3 μg/mL. (Inset) Normalized PA amplitude at 500 nm with increasing [CFZ-HCl NP] on a normal scale to show the approximate linearity between [CFZ-HCl NP] and the PA amplitude, at least up to a concentration of 0.3 mg/mL when signal saturation starts to occur.

After confirming that the CFZ-HCl NP accumulated at a higher concentration in the prostates of TRAMP mice, we identified if this increased accumulation could be detected via PA imaging. Since one of the main concerns for detecting the CFZ-HCl NP was its potential overlap in absorption spectra with blood, specifically with HbO_2_ and Hb (Fig 3A), we first tested the minimum detectable concentration of CFZ-HCl NP in the presence of blood. Here, a concentration of 10% blood by volume was used to mimic physiological conditions [45]. A wavelength of 500 nm was used because it was close to the peak absorption of CFZ-HCl NP, and it is also at a trough and isosbestic point of HbO_2_ and Hb. By looking at the increase in PA signal amplitude at 500 nm with increasing CFZ-HCl NP concentrations, we were able to detect a change in PA signal with a concentration of up to 3 μg/mL of CFZ-HCl NP (Fig 3B). Assuming that 1 g of the prostate organ has a volume of approximately 1 mL (with 1 g/mL being the density of water), CFZ-HCl NP was found to accumulate in the prostates of TRAMP mice with an average concentration of 60 μg/mL, as determined from the result in Fig 2C. This accumulation concentration is more than an order of magnitude above the detection threshold of 3 μg/mL. Hence, we concluded that the accumulation of CFZ-HCl NP in the prostates of TRAMP mice will be detectable via PA imaging.

### CFZ-HCl NP imaging via PA multiple wavelength unmixing

**Fig 4:**
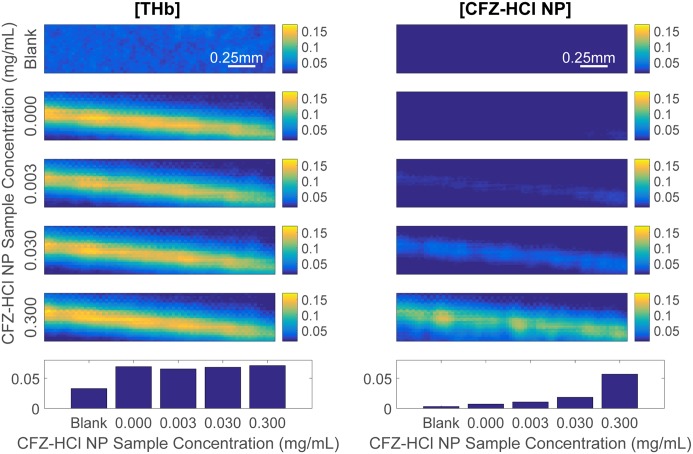
Two-wavelength unmixing of CFZ-HCl NP from blood. A mixture of different concentrations of CFZ-HCl NP (shown on the y-axis) with 10% blood by volume in a tube was used. The two columns indicate the isolated contributions of THb and CFZ-HCl NP to the PA signal for each sample. Color bars only indicate the relative concentrations and not the actual concentrations. The last row shows the quantified relative concentrations for all the samples. “Blank” indicates a tube filled with water.

Next, we verified if we would be able to distinguish the CFZ-HCl NP signal from the blood signal. To do this, we used the multiple wavelength unmixing method to decouple the CFZ-HCl NP signal from that of the blood. This method involves using multiple measurements at different laser wavelengths to isolate the contributions of each chromophore to the PA signal [46, 47]. In this study, only two wavelengths were used to identify the individual contributions of CFZ-HCl NP and total hemoglobin. Here, we observed that the CFZ-HCl NP signal could be distinguished from the THb signal (Fig 4). This was demonstrated by the fact that we saw no change in the measured THb concentration after the deconvolution while the measured concentration of CFZ-HCl NP increased as expected. The blank solution (water) also showed very low THb and CFZ-HCl NP signals. However, there was a difference in the background relative signal of THb and CFZ-HCl NP which was attributed to the fact that the tubing used to hold the samples generates a small PA signal on its own, leading to some small systematic errors in the measurement. Hence, we demonstrated that the multiple wavelength unmixing method worked as intended.

### *Ex vivo* PA imaging of prostate samples

**Fig 5:**
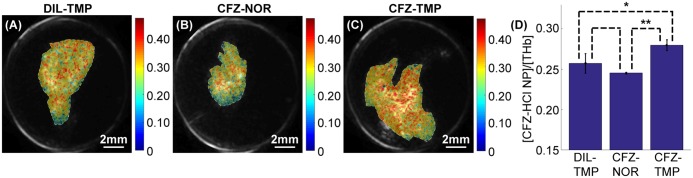
*Ex vivo* PA imaging results. CFZ-HCl NP signal normalized to the THb signal for prostates of (A) diluent injected TRAMP mice (DIL-TMP), (B) CFZ-HCl NP injected normal mice (CFZ-NOR), and (C) CFZ-HCl NP injected TRAMP mice (CFZ-TMP). Color bars indicates the CFZ-HCl NP signal normalized to the THb signal as determined by the two-wavelength unmixing. (D) Mean normalized CFZ-HCl NP signal for each group within the organ boundaries as delineated by the US image (shown in the representative images as a white dotted line). * p-value < 0.05, ** p-value < 0.01.

After validating that decoupling the contributions of CFZ-HCl NP and blood could be achieved by multiple wavelength unmixing via the experiment on tube samples, we then performed *ex vivo* PA imaging of the mouse prostates. Here, we compared the same 3 groups of mice shown in Fig 2. Fig 5A–5C show the normalized CFZ-HCl NP signal over the blood signal for representative prostates in the 3 separate groups. The CFZ-HCl NP signal was normalized to the blood signal to help reduce any inaccuracies due to the difference in size of the prostates, as detailed in the methods. Fig 5D shows the normalized mean CFZ-HCl NP signal to the mean THb signal for all the prostates in each of the 3 groups. We observed that there was a significantly higher CFZ-HCl NP signal in the prostates of CFZ-HCl NP injected TRAMP mice compared to the other two groups, which matched the results shown in Fig 2. Furthermore, there was no significant difference between the diluent injected TRAMP prostates and the CFZ-HCl NP injected normal prostates. Thus, we demonstrated that PA imaging can be used to detect the differences in accumulation of CFZ-HCL NPs in the prostates in an *ex vivo* setting.

## Discussion

While the multiple wavelength unmixing technique helps to mitigate the interference of endogenous chromophores, it is not a perfect technique, and there will always be background noise from chromophores that are not accounted for in the algorithm. For example, the imaging results in Fig 5 showed a strong background signal for CFZ-HCl NP in the absence or low concentration of the drug. In addition, there appeared to be a slightly higher CFZ-HCl NP signal in the DIL-TMP group compared to the CFZ-NOR group, in contrast with the histology results where the reverse was true. Unlike the samples used in Fig 4, other chromophores such as myoglobin (and its many different forms) could also be present in the prostate, contributing to some inaccuracies in the CFZ-HCl NP signal. If these chromophores could be identified and accounted for, with more PA measurements made at additional wavelengths, the sensitivity of the PA imaging technique towards CFZ-HCl NP could be improved. In addition, the fluence distribution in the prostate tissues is assumed to be wavelength-independent, which is typically a good assumption for near-surface PA imaging, less so with increasing imaging depth [46, 48]. Addressing these two limitations in the current approach would improve the sensitivity of PA imaging towards the detection of CFZ-HCl NP, allowing for even more precise and accurate detection. The ability to detect and distinguish smaller concentrations of CFZ-HCl NP will allow for many new possibilities using this technique, such as the study of prostate cancer progression in its early stages, where CFZ-HCl NP accumulation in the prostate is expected to be small. Additionally, there are concerns that normalization of the CFZ-HCl NP to the THb signal would reduce the sensitivity of PA imaging to detect CFZ-HCl NPs due to increasing vasculature in the prostate tumor with cancer progression. While this may be true, we believe that the difference in accumulation of CFZ-HCl NPs between cancerous and non-cancerous prostates (as shown in Fig 2C) will far outweigh any variations in tumor vasculature.

It should also be noted that there are other PA contrast agents that can be used in the near-infrared spectrum range (e.g. indocyanine-green and gold nanorods) [49, 50], which would lead to less endogenous chromophore interference. However, none of them come with the intrinsic benefit of CFZ-HCl NP, which specifically accumulates in tissue macrophages without the need for external targeting agents. Since TAMs are prevalent in most forms of cancer, this drug could potentially be used in the diagnosis of other forms of cancer as well. Furthermore, most of the other contrast agents are not clinically approved and are still limited to the research field [49]. CFZ, on the other hand, is an FDA-approved drug with minimal harmful side-effects [40], which makes it more likely to be translated for clinical use. Therefore, we expect the benefits of using CFZ-HCl NP as a diagnostic contrast agent to outweigh the higher signal interference with endogenous chromophores compared to other known contrast agents.

Besides the limitations mentioned above, there are also other challenges for translating this into a practical probe for *in vivo* monitoring of prostate cancer development and staging, such as the imaging depth needed for imaging prostates *in vivo* due to the prostate’s anatomical location both in animals and humans. Due to the CFZ-HCl NPs low absorption wavelength, deep imaging depths with the nanoparticle may be limited. However, there is ongoing research in this field, where potential solutions to this include the development of a minimally invasive needle-based PA imaging system that can be inserted close to the prostate [18, 51–53]. This helps to bring both the light source and ultrasound transducer close to the prostate, significantly reducing the absorption of the light signal from other chromophores as well as the attenuation of the ultrasound signal in the body.

In conclusion, we have shown that IV injected CFZ-HCl NP accumulates at a higher concentration in cancerous prostates than in normal prostates within 24 hours post injection in a TRAMP mouse model. In the presence of blood, one of the main endogenous chromophores in the body, we were able to isolate the signal of CFZ-HCl NP using two-wavelength unmixing PA imaging. Furthermore, we were able to apply this technique in PA imaging to detect the higher accumulation of CFZ-HCl NP in the cancerous prostates compared to the normal prostates. Hence, we believe that differences in the interaction of CFZ-HCl NP with normal vs cancerous prostates, together with PA imaging, could lead to the development of a noninvasive technique for the detection and monitoring of prostate tumor induction and progression in an animal model of the disease, while providing useful information to facilitate the design of next generation imaging probes for the development of noninvasive diagnosis and staging of prostate cancer in humans.

## Supporting information

S1 File NC3Rs ARRIVE guidelines checklist. This file contains additional information on the appropriate use of animals in this research study.
(PDF)
